# State-dependent inter-network functional connectivity development in neonatal brain from the developing human connectome project

**DOI:** 10.1016/j.dcn.2024.101496

**Published:** 2024-12-12

**Authors:** Zhiyong Zhao, Ruolin Li, Yihan Wu, Mingyang Li, Dan Wu

**Affiliations:** a Department of Radiology, Children’s Hospital, Zhejiang University School of Medicine, National Clinical Research Center for Child Health, Hangzhou, China; b Department of Radiology, Children’s Hospital of Philadelphia, Philadelphia, USA; c Department of Biomedical Engineering, Johns Hopkins University, USA; d Key Laboratory for Biomedical Engineering of Ministry of Education, Department of Biomedical Engineering, College of Biomedical Engineering & Instrument Science, Zhejiang University, Hangzhou, China

**Keywords:** Neonate, Resting-state network, Dynamic functional connectivity, State-dependent, Preterm, DHCP

## Abstract

Although recent studies have consistently reported the emergence of resting-state networks in early infancy, the changes in inter-network functional connectivity with age are controversial and the alterations in its dynamics remain unclear at this stage. This study aimed to investigate dynamic functional network connectivity (dFNC) using resting-state functional MRI in 244 full-term (age: 37–44 weeks) and 36 preterm infants (age: 37–43 weeks) from the dHCP dataset. We evaluated whether early dFNC exhibits age-dependent changes and is influenced by preterm birth. Gestational age (GA) and postnatal age (PNA) showed different effects on variance of FNC change over time during fMRI scan in resting-state networks, especially among high-order association networks. These variances were significantly reduced by preterm birth. Moreover, two states of weakly-connected (State Ⅰ) and strongly-connected (State Ⅱ) FNC were identified. The fraction window and dwell time in State Ⅰ, and the transition from State Ⅱ to State Ⅰ, all showed significantly negative correlations with both GA and PNA. Preterm-born infants spent a longer time in the weakly-connected state compared to term-born infants. These findings suggest a state-dependent development of dynamic FNC across brain networks in the early stages, gradually reconfiguring towards a more flexible and dynamic system with stronger connections.

## 1 Introduction

Neonatal brain development has received much attention in recent years due to its rapid growth and vulnerability to environmental influences (Gao et al., 2015). During this period, brain function undergoes tremendous development based on the earlier maturation of brain structures (Liu et al., 2021, Ouyang et al., 2019). Resting-state networks (RSNs) (Damoiseaux et al., 2006, Mantini et al., 2007) consist of spatially distinct brain regions that exhibit a low-frequency temporal coherence, including primary networks (e.g., sensorimotor, visual, auditory) and high-order networks (e.g., default mode, executive control), which have been demonstrated to be present and detectable in neonates (Fransson et al., 2007). The functional connectivity (FC) within RSNs is usually examined to characterize early brain functional development in infants. Intra-network FC is considered to increase with normal development (Li et al., 2023, Zhang et al., 2019), and its decrease may indicate a developmental delay and an increased vulnerability to neuropathological attacks (Menon, 2013). Moreover, inter-network FC, namely functional network connectivity (FNC), is another important characteristic of brain function, reflecting synchronization and interaction among RSNs, which has been focused in numerous studies of early brain development (Gao et al., 2015, Gao et al., 2013). However, it remains inconsistent that the FNC increases (Gao et al., 2015) or decreases (Damaraju et al., 2014, Zhang et al., 2017) with age during early brain development in previous reports.

Conventional functional connectome analysis abovementioned assumes that the FC is static during the scan, and it does not account for the dynamic variation of regional communication over time. However, accumulating evidence has demonstrated that the brain dynamically integrates, coordinates, and responds to internal and external stimuli across multiple time scales (Hutchison et al., 2013, Zalesky et al., 2014, Braun et al., 2015). The dynamic properties of FC among functional networks or brain regions are closely linked to primary and cognitive functions (Calhoun et al., 2014, Chyl et al., 2021, Erb et al., 2013, Hellyer et al., 2015). Therefore, characterizing FC dynamics during normative development could help to deepen our understanding of functional flexibility and behavioral changes in infants. Several functional neuroimaging studies in neonates have reported significant age-related changes in temporal variability of regional FC (Gao et al., 2022, Wen et al., 2020). However, few studies have explored the development of FNC among RSNs and focus on brain state dynamics during fMRI scan, which have shown age-related changes from late childhood to young adulthood (Allen et al., 2014, Marusak et al., 2017, Medaglia et al., 2018) and during infancy (França et al., 2022). Therefore, the understanding of early brain developmental process in FNC dynamics remains in the dark, and the similar delineation of developmental FNC during this pivotal period is warranted.

Previous researches demonstrate that the variability in early brain system development is related to multiple sources of prenatal and postnatal adversity and biological stress mediators (Graham et al., 2021). Therefore, gestational age (GA, time elapsed between the first day of the last menstrual period and the day of delivery) and postnatal age (PNA, time elapsed from birth), associated with intra-uterine environment and extra-uterine exposure respectively, may have distinct effects on early brain development and reflect different time courses for neuronal development (Arimitsu et al., 2022). Moreover, a recent study indicate that white matter myelination occurs more rapidly before than after birth in preterm infants (Grotheer et al., 2023). The premature exposure (smaller GA) can lead to a decelerated developmental trajectory in preterm infants compared to term-born infants (Eyre et al., 2021, Fenn-Moltu et al., 2023), while more prolonged exposure (larger PNA) may result in accelerated maturation patterns in preterm infants (Pelc et al., 2022). Thus, the premature brain may follow a different developmental trajectory compared to full-term counterparts, both before and after birth. However, both GA and PNA were usually mixed into the age (postmenstrual age, PMA) in previous studies. Therefore, this study employed the dynamic FNC (dFNC) analysis to investigate the relationships between temporal variability of FNC as well as brain state features (fractional windows, mean dwell time, and transition) and GA/PNA in full-term infants. Moreover, we compared differences in the FNC measures between preterm and term infants. Conventional static FNC (sFNC) is too simplistic to capture the complete representation of FNC evolution during fMRI scan, therefore, we were also interested in exploring differences between the sFNC and dFNC during early development. We hypothesized: (1) static and dynamic FNC differed in spatiotemporal patterns; (2) dFNC development in neonatal brain was state-dependent; (3) preterm birth had a significant effect on dFNC.

## 2 Methods

### 2.1 Subjects

The data used in this study were obtained from the second release of the dHCP, an observational and cross-sectional Open Science program approved by the UK National Research Ethics Authority (14/LO/1169) and informed written consent given by the parents of all participants.

In the dHCP, term-born infants who were clinically well were recruited from the postnatal wards, while preterm-born infants were recruited from neonatal units and postnatal wards. Infants with severe compromise at birth requiring prolonged resuscitation, diagnosed chromosomal abnormalities, contraindications to MRI scan, or clinically significant brain injury were excluded from the study (Denisova, 2019, Vanes et al., 2023). Only term-born infants scanned between 37 and 44.5 weeks, or preterm-born infants scanned between 37 and 43.5 weeks were considered for inclusion. Thus, we initially screened 408 individuals with structural and functional MRI data. Then, 77 infants were excluded due to a low radiology score ≤ 2, (i.e., possible clinical significance and poor quality anatomical data), and 51 infants were excluded due to excessive head motion (see FMRI preprocessing and Head motion analysis). Finally, 280 infants, including 244 term-born infants (136 males) and 36 preterm-born infants (23 males), entered into final analysis.

### 2.2 FMRI data acquisition

MRI data were acquired at a 3 T Philips Achieva system with a 32-channel receive coil. All infants were in natural sleep without sedation. The rs-fMRI was acquired using a multislice gradient-echo echo planar imaging (EPI) sequence with multiband excitation to obtain high temporal resolution BOLD fMRI. The scan parameters were as follows: repetition time (TR) = 392 ms, echo time (TE) = 38 ms, flip angle = 34°, voxel size = 2.15 × 2.15 × 2.15 mm^3^. This scan session lasted for 15 minutes and 3 seconds, resulting in 2300 volumes.

### 2.3 FMRI preprocessing

The rs-fMRI data were preprocessed using an optimized pipeline for each individual by dHCP group (Fitzgibbon et al., 2020), including slice-to-volume motion correction, susceptibility distortion correction, bespoke ICA-based denoising, and an automated quality control framework. Subsequently, we registered the averaged fMRI image to a 40-week T2 template (with a spatial resolution of 2 × 2 × 2 mm^3^) using the FLIRT boundary-based registration method for each individual, which generated a transform and was applied to all functional images. We visually checked registered images and corrected them for each subject. Next, we removed eight nuisance variables, including six head motion parameters (three translation and three rotation parameters), white matter and cerebral-spinal fluid signals, using a multivariate linear regression analysis. Finally, the functional images were spatially smoothed using a Gaussian kernel with a full width at half-maximum (FWHM) of 4 mm and were temporally filtered (0.01–0.1 Hz).

### 2.4 Head motion analysis

The fMRI preprocessing pipeline included the head motion correction, and the natural sleep MRI scan protocols helped reduce motion in unsedated infants. However, considering the significant effect of head motion on resting-state BOLD signal, in order to minimize potential bias, a further conservative approach was taken, in which a continuous subsample of the data with the lowest motion (approximately 70 %) was selected and those with a high level of motion were excluded (Eyre et al., 2021).

Specifically, we calculated the mean frame-wise displacement (FD) using the formula of Power et al. (2012), which combined translational and rotational scan-to-scan displacement based on three translations (x, y, z axes) and three rotations (pitch, yaw, roll) parameters obtained during realignment for each participant. Motion outliers were defined as volumes with FD > 1.5 interquartile range (IQR) above the 75th percentile according to a previous study (Fitzgibbon et al., 2020). The subjects with more than 160 motion-outlier volumes (10 % of the cropped dataset) or their mean FD > 0.5 mm (Liu et al., 2023), were excluded entirely. Then, for each subject, the continuous 1600 volumes with the minimum number of motion-outlier volumes were identified and used for the subsequent analyses. This allowed us to minimize the potential effect of different states of arousal even after data denoising. Finally, the head motion was also included as a covariate in all subsequent statistical analyses.

### 2.5 Resting-state network analysis

Given that the association RSNs may exhibit distinct patterns in infants at different PMA. Therefore, group-level analysis should be performed for the subjects with the same PMA but not all subjects in infant study, differing from ICA analysis in the adult studies. The RSNs were defined by a group-level independent component analysis (ICA) in the term-born infants scanned at 43.5–44.5 week PMA (n = 27) using GIFT toolbox (http://icatb.sourceforge.net). The ICA was conducted with a dimensionality set at 30, balancing robustness and interpretability (Toulmin et al., 2015). The output comprised 30 group-average components, and each of them included an independent spatial map and its corresponding time series. The spatial maps were visually inspected, and spatial correlation coefficients between the selected components and the templates reported (Smith et al., 2009) were computed and ranked, with manual classification as either RSN or noise, according to the guidelines reported by Fitzgibbon et al. (Fitzgibbon et al., 2020). Then, we used the group-level spatial maps to generate subject-specific maps and obtain the time series via a dual regression using FSL melodic (https://fsl.fmrib.ox.ac.uk/fsl/docs/#/resting_state/dualregression) (Nickerson et al., 2017) in 217 term-born infants scanned at 37–43.5 weeks PMA and 36 preterm-born infants. Specifically, we first performed multiple regression analyses for each subject to regress the group-level RSN spatial maps (as spatial regressors) into the subject’s 4D space-time dataset, resulting in a set of subject-specific time series for each group-level spatial map. Subsequently, we performed another multiple regression analysis to regress these time series (as temporal regressors) into the same 4D dataset, resulting in a set of subject-specific spatial maps for each group-level spatial map. Finally, we obtained individual RSNs and their time series for each infant.

### 2.6 Dynamic functional network connectivity analysis

We calculated the static FNC by using Pearson correlation between the time series of any paired RSNs for each infant before dFNC analysis. Then, we assessed the dFNC using a sliding window approach (Hindriks et al., 2016) within the Dynamic BC toolbox (Liao et al., 2014), which has been proven to work well for evaluating dynamic properties of regional neural activity (Cui et al., 2020). The analysis of functional connectivity state occurrences revealed persistence of states for approximately 5–10 s, and thus the sliding window approach requires a window length of at least about 20 seconds to observe state transitions (Preti et al., 2017). To enhance the accuracy of k-means clustering by obtaining the connectivity matrices as many as possible. Here, we selected a window length of 50 TR (19.6 s) for dFNC analysis, consistent with parameters used in previous studies (Cao et al., 2022, Li et al., 2020, Preti et al., 2017), with a step of 2 TRs (0.784 s), the time series of each RSN were divided into 78 windows, and the FNC matrix for each window and the variance of FNC across all windows were calculated for each subject.

Next, we used k-means clustering methods to estimate recurring FNC patterns (states) from 78 connectivity matrices in the term-born infants scanned at 43.5–44.5 week PMA. To estimate the optimal number of clusters, we performed a cluster number validity analysis (gap and silhouette statistic) on the subsampling windows while varying the number of clusters from 2 to 4. Based on the gap statistic (Tibshirani and Hastie, 2001) and the silhouette statistic (Peter, 1987), which measure the similarity between windows in the same cluster compared to similarity with windows in a different cluster, we determined the optimal number of clusters to be two (k = 2). Thus, we obtained two group-level states (state Ⅰ and Ⅱ) of the dFNC. After that, we first categorized 78 FNC matrices of each full-term infant scanned at 37–43.5 weeks PMA and each preterm infant into two states based on their similarity with the two group-level cluster centroids using Pearson correlation. This generated a state transition vector for each infant, representing changes in dFNC state over time. Then, all state Ⅰ-like or state Ⅱ-like FNCs were averaged as the individual-level state Ⅰ or state Ⅱ, respectively. To investigate the temporal nature of dFNC states, we examined three variables based on the state transition vector: fractional windows, mean dwell time, and number of transitions. Fractional window denotes the percentage of time spent in each state. Mean dwell time represents the average duration of time that the participant remained in a particular state before transitioning to another state. The number of transitions counts the number of times the state changed from one to the other, indicating the level of stability over time.

### 2.7 Statistical analysis

We used general linear models (GLM) to perform cross-subject analysis and investigate the effects of GA at birth, PNA and preterm birth on FNC measures (Table 1). In order to reduce ex-utero environmental influences due to the time duration between birth and the scan, for GA-dependent analysis, we selected 155 term-born neonates who underwent MRI within one week of birth with sex and motion as covariates. Considering that the effect of PNA on infant development varies depending on the gestational weeks at birth (Arimitsu et al., 2022), we selected 74 term-born neonates with a fixed 40-week GA, and used sex and motion as covariates. Moreover, we compared the differences of dFNC measures between 36 preterm-born neonates and 36 matched term-born neonates, with PMA, sex, and motion as covariates. We performed all analyses at the three levels of whole brain, each network and each connection. Here, the FNC for each RSN was defined as the averaged connection between itself and other RSNs. All p-values were corrected using the family-wise error (FWE) method (p < 0.05) to control false positive discoveries.

**Table 1:** Information of three data subsets.

Group	Regression model	Selection criteria	Sample size	PMA(week)	GA (week)	PNA (week)	Sex (M:F)	Head motion(mm)
FNC ∼ GA	FNC ∼ β0 + β1 *GA + β2 *Sex + β3 *Motion	PNA< 1 week	155	40.29 ± 1.29	39.93 ± 1.32	0.36 ± 0.23	92:63	0.20 ± 0.08
FNC ∼ PNA	FNC ∼ β0 + β1 *PNA + β2 *Sex + β3 *Motion	GA = 40 week	74	41.53 ± 1.44	40.08 ± 0.29	1.45 ± 1.42	43:31	0.21 ± 0.11
Preterm vs Term	FNC ∼ β0 + β1 *group+ β2 *PMA + β3 *Sex + β4 *Motion	PMA match	36 vs 36	P = 0.95	P < 0.001	P < 0.001	P = 0.96	P = 0.39

M:F: male:female.

## 3 Results

### 3.1 Resting-state networks

**Fig. 1:**
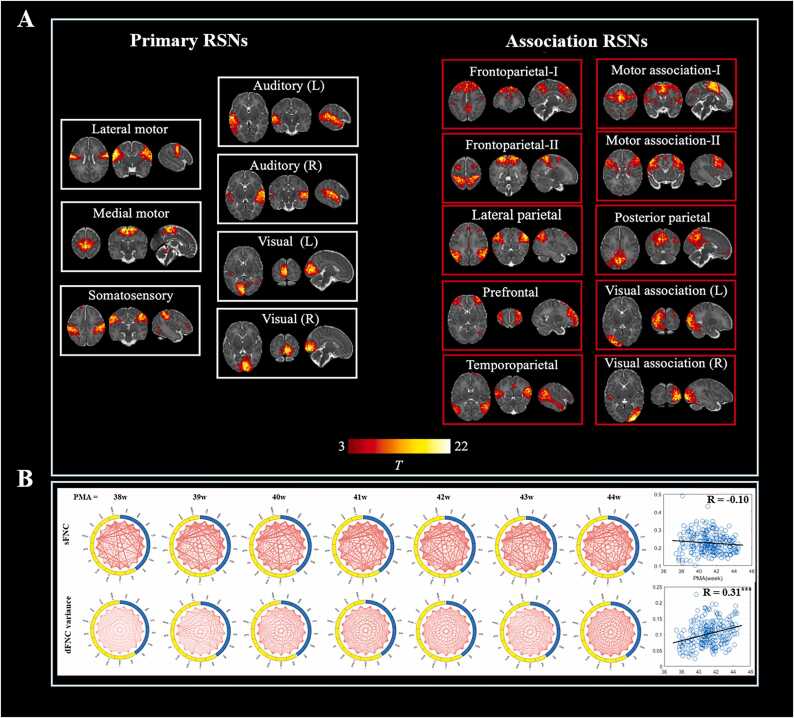
**Resting state networks identified by group independent component analysis.** Panel A shows seventeen RSNs derived from group ICA in 27 term-born infants scanned at 43.5–44.5 weeks PMA using one-sample t test. Example axial, coronal, and sagittal slices for spatial patterns in seven primary (white rectangles) and ten association (red rectangles) networks, thresholded at T > 3 (one-sample *t*-test for each RSN) and overlaid on a T2-weighted template. Panel B shows averaged connection patterns of static FNC and variance of dFNC in term infants at each PMA (sample size for 38–44w: N = 20, 28, 30, 60, 48, 31 and 27). In each chord diagram, the yellow and blue of the nodes represent association and primary networks, respectively. The scatter plots represent correlations between averaged connections/variances in whole brain and PMA across all full-term infants. * ** : p < 0.001.

Seventeen RSNs were identified using group ICA in term-born infants with 44-week PMA, including seven primary networks (motor, somatosensory, auditory and visual) and ten high-order association networks (frontoparietal, prefrontal, parietal, temporoparietal, motor association, and visual association). The spatial maps of all components were displayed in Figure S1. Fig. 1A illustrates the spatial patterns of the cortical regions within each RSN. As shown in Fig. 1B, with increasing PMA, the group-averaged variance of dFNC gradually increased, whereas the sFNC showed no an evident change.

### 3.2 Age-dependent change in FNC

**Fig. 2:**
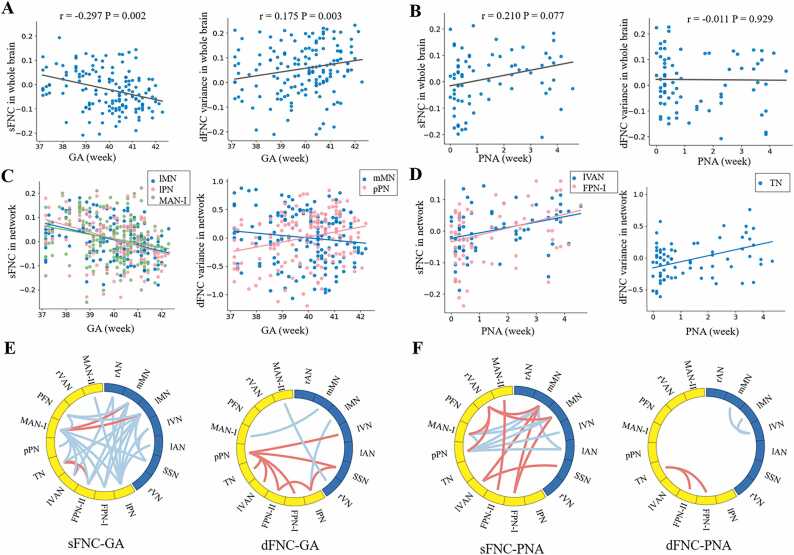
**The effect of age on FNC among RSNs.** The three rows in the figure show changes in both sFNC and variance of dFNC with GA and PNA at the whole brain level (which were based on the average of all edges) (A-B), network level (C-D) and connection level (E-F), respectively. Here, the FNC for each RSN was defined as the averaged connection between itself and other RSNs. In each chord diagram, the yellow and blue of the nodes represent association and primary networks, respectively. Red and blue lines represent positive and negative correlations, respectively. mMN/lMN: medial/lateral motor network; SSN: somatosensory network; rVAN/lVAN: right/left visual association network; FPN: frontoparietal network; pPN/lPN: posterior/lateral parietal network; TN: temporoparietal network; MAN: motor association network; PFN: prefrontal network; rAN/lAN: right/left auditory network; rVN/lVN: right/left visual network.

At the whole-brain level, the sFNC and variance of dFNC in term-born infants showed negative and positive correlations with GA respectively (Fig. 2A), but neither showed significant correlations with PNA (Fig. 2B). At the network level, the sFNC in the lateral motor network (lMN), motor association network (MAN-I) and lateral parietal network (lPN) negatively correlated with GA, while the variance of dFNC in the medial motor network (mMN) and posterior parietal network (pPN) negatively and positively correlated with GA, respectively (Fig. 2C). The sFNC in the left visual association network (lVAN) and frontoparietal network (FPN-Ⅰ), and the variance of dFNC in the temporoparietal network (TN) were positively correlated with PNA (Fig. 2D).

At the connection level, the sFNC showed predominantly negative correlations with GA but positive correlations with PNA, while the variance of dFNC displayed more positive correlations with GA than PNA, especially among high-order networks (Fig. 2E-F). These findings were validated by the GLM analysis (FNC ∼ β0 + β1 *GA + β2 *Sex + β3 *Motion) in 244 full-term infants (Table S1 and Fig. S2). Collectively, we found different effects of GA and PNA on FNC, suggesting a developmental impact on large-scale functional networks during early infancy. Also, the dFNC revealed distinctive development-related FNC changes compared with conventional sFNC, thereby complementing existing research on FNC development.

### 3.3 Brain states of FNC among RSNs and their changes with age

**Fig. 3:**
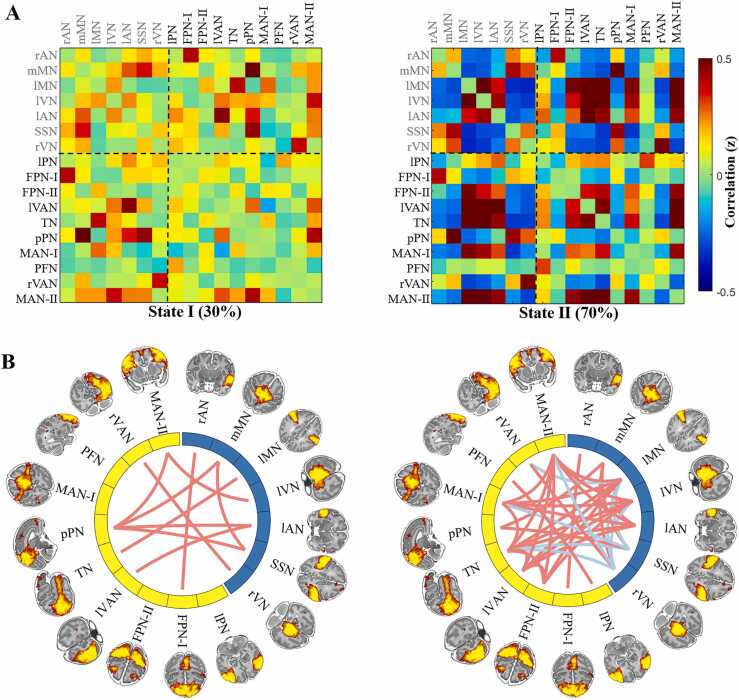
**Results of the clustering analysis in the term-born infants scanned at 43.5–44.5 weeks PMA.** A: Cluster centroids for each state. The percentage of total occurrences is listed for each state. The colorbar represents the z value of the correlations. Red and blue colors represent positive and negative correlations, respectively. The labels with gray font represent primary networks. B: The strongest 10 % significant connections of each state.

The clustering analysis divided dynamic FNC in full-term neonates at 43.5–44.5 weeks PMA into two distinct connectivity states: weakly-connected (State I) and strongly-connected (State II) states (Fig. 3). Among them, State II (70 %) occurred more frequently than State I (30 %) during an MRI scan, implying that the brain prefers to stay in the strongly-connected state during early development.

**Fig. 4:**
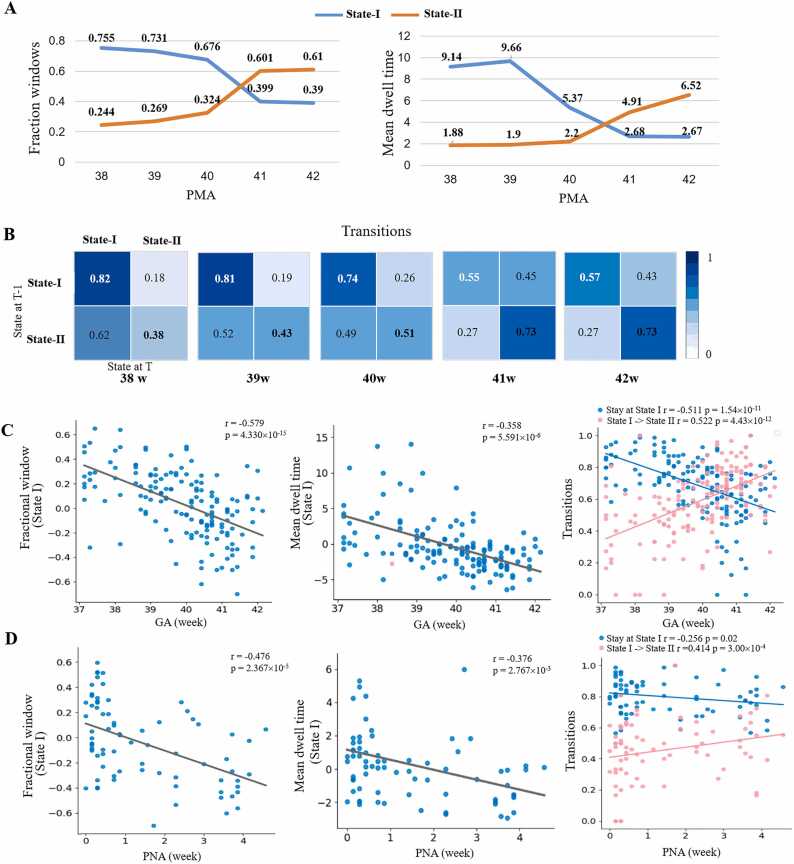
**Temporal properties of FNC state in full-term infants.** A-B represent fractional windows, mean dwell time, and transition between State I and State II changing with PMA increase at the group level. C-D show significant correlations between three temporal variables and GA/PNA at the individual level.

At the group level, as PMA increased, we observed a decrease in both fractional window and dwell time in State I, while these metrics increased in State II (Fig. 4A). Additionally, the transition probability from State I to State II also showed an increase (Fig. 4B) in full-term infants. At the individual level, the three temporal variables in State I all showed significant negative correlations with both GA (Fig. 4C) and PNA (Fig. 4D), while transition probability from State I to State II showed significant positive correlations. These findings indicate a developmental shift in FNC patterns among RSNs from weakly-connected to strongly-connected states during early infancy, irrespective of GA or PNA.

### 3.4 Effect of preterm birth on FNC

**Fig. 5:**
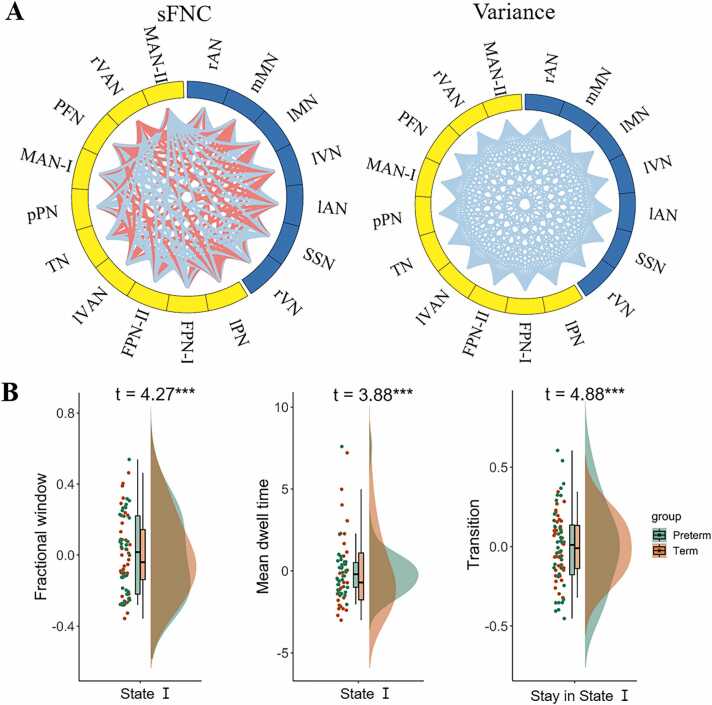
**The effect of preterm birth on FNC.** In each chord diagram of panel A, the yellow and blue of the nodes represent association and primary networks respectively; the red and blue of lines represent increased or decreased FNC in preterm compared to term, respectively. In panel B, positive t values demonstrate that preterm group has higher values compared to term group.

Compared with term-born neonates, preterm-born neonates showed a smaller variance of dFNC (*P* < 0.05) but no significant difference in the sFNC at the whole-brain level (*P* > 0.05). At the network level, preterm-born neonates showed increased sFNC in the PFN and decreased sFNC in the medial MAN-Ⅱ, along with decreased variance of dFNC in all RSNs (Table 2). Regarding level, preterm-born neonates displayed significant increases or decreases in the sFNC and significant decreases in the variance of dFNC (Fig. 5A). Moreover, the preterm group showed increased fractional windows, dwell time and transition in State I compared with the term group (Fig. 5B), suggesting that the FNC in the preterm-born infants may prefer to stay in the weakly-connected state.

**Table 2:** Significant differences between preterm- and term-born infants in FNC of each RSN.

**Network**	**t**	**p**	**Cohen’s d**	**Effect size**
*sFNC*				
Prefrontal network	5.44	8.00 × 10–7	1.30	0.55
Motor association network (Ⅱ)	−3.23	1.88 × 10–3	−0.77	0.36
*dFNC*				
Right auditory network	−5.77	2.24 × 10–7	−1.38	0.57
Medial motor network	−6.15	4.91 × 10–8	−1.47	0.59
Lateral motor network	−5.73	2.60 × 10–7	−1.37	0.57
Left visual network	−6.06	7.06 × 10–8	−1.45	0.59
Left auditory network	−5.95	1.09 × 10–7	−1.42	0.58
Somatosensory network	−6.95	1.85 × 10–9	−1.66	0.64
Right visual network	−5.97	9.86 × 10–8	−1.43	0.58
Lateral parietal network	−4.69	1.37 × 10–5	−1.12	0.49
Frontoparietal network (Ⅰ)	−5.44	8.11 × 10–7	−1.30	0.55
Frontoparietal network (Ⅱ)	−5.49	6.60 × 10–7	−1.31	0.55
Left visual association network	−6.17	4.54 × 10–8	−1.47	0.59
Temporoparietal network	−6.44	1.51 × 10–8	−1.54	0.61
Posterior parietal network	−6.24	3.31 × 10–8	−1.49	0.60
Motor association network (Ⅰ)	−4.93	5.64 × 10–6	−1.18	0.51
Prefrontal network	−5.59	4.57 × 10–7	−1.34	0.56
Right visual association network	−5.61	4.12 × 10–7	−1.34	0.56
Motor association network (Ⅱ)	−5.54	5.51 × 10–7	−1.32	0.55

## 4 Discussion

The present study investigated the dynamic functional connectivity among resting-state networks and how it changes with age in infant brains. The results supported our initial hypothesis, and demonstrated that the FNC state displayed a short transition from weakly-connected to strongly-connected state after birth, which was affected by preterm birth. Moreover, dynamic and static FNC revealed different spatiotemporal changes with age in connection patterns. Therefore, age-related alterations in inter-network functional connectivity may exhibit a significant temporal variance during early brain development.

### 4.1 Spatial-temporal developmental patterns in full-term infants

We found that the variance of dFNC showed a positive correlation and sFNC showed a negative correlation with GA at the whole brain level, but neither showed a significant association with PNA. This may suggest a unique pattern of global dynamics at birth, which is independent of ex-utero life exposure, supporting cognitive development in immature individuals before birth (Hellyer et al., 2015). Our results extended prior findings showing altered global dynamic connectivity after birth (França et al., 2022) and later in childhood (Padilla et al., 2020), indicating that changes in whole-brain connectivity dynamic had occurred at birth.

We also revealed an age-dependent functional development of either sFNC or variance of dFNC in a spatially heterogeneous manner. FNC changes in primary networks (e.g., motor network) and posterior association networks (e.g., parietal network) significantly correlated with GA, while those in anterior association networks (i.e., frontoparietal and temporoparietal networks) significantly correlated with PNA. These findings suggest that the functional development of primary networks may be primarily affected by intrauterine, rather than extrauterine experience, as they have been reported to be mature and adult-like pattern before birth (Wen et al., 2020). The development of FNC in high-order networks progresses from the parietal lobe to the frontal and temporal lobes before and after birth, indicating a developmental sequence of brain functions from posterior to anterior (Gao et al., 2015). The previous studies including both fetuses and infants have reported that FC in primary sensorimotor networks is established at birth, whereas FC in high-order cortical networks undergoes a more protracted development associated with PNA during infancy (Gao et al., 2015, Van den Heuvel and Thomason, 2016).

Additionally, at the connection level, we found that the variance of dFNC increased among high-order association networks but decreased among primary networks with both GA and PNA. These findings indicate that association networks exhibit increasing temporal variability, possibly due to the earlier maturation of primary regions compared to high-order regions, as suggested by numerous static FC studies (Cao et al., 2017, Gilmore et al., 2018) and structural studies (Huang et al., 2015, Kostović et al., 2014). The high-order networks shows such a more frequent and more active information communication with other RSNs compared to primary networks, possibly supporting early complex brain function development (Cole et al., 2013). The decreasing flexibility in primary networks may indicate that the primary sensory functions require more stable FC to maintain a stable and robust bottom-up information flow to high-level functional networks (Wen et al., 2020). Collectively, our findings support the existence of spatial-temporal functional development in flexibility and stability of FNC among the RSNs during early infancy.

### 4.2 Brain states switched shortly in full-term infants after birth

During early infancy, as the brain matures, the number of possible functional network configurations (corresponding to RSNs) based on the earlier maturation of brain structures increases to support emerging high-order cognitive functions (Wen et al., 2020), such as self-awareness, spatial attention, and working memory. This requires cooperation among multiple functional networks, since they are largely incomplete in the infant brain (Gao et al., 2015, Gao et al., 2015). Moreover, the brain networks usually has a more variable system at the early stage, to ensure the information processing capacity among the increasing number of brain states (Deco et al., 2009, McIntosh et al., 2010), since a larger variance indicates richer connection patterns possibly underlying more frequent information exchange among different networks (Dong et al., 2019). Therefore, the FNC with age in neonatal brain would tend to develop a pattern with strong connections among RSNs.

Here, we identified two transient states showing weakly- and strongly-connected FNC among the RSNs in the newborn brain at term equivalent age, respectively. With the increase of GA or PNA, changes in fractional time, dwell time and transition of states demonstrated a preference for the brain to stay in the strongly-connected state, implying a more efficient and closer information communication among RSNs to facilitate functional integration of the whole brain during development (Grayson and Fair, 2017, Zhang et al., 2019). Similarly, recent studies revealed state-dependent functional connectome development in neonatal brains, in which the modular architecture of connectomes spontaneously reconfigures over time (França et al., 2022), affecting neurodevelopmental and behavioral outcomes. Prior studies in adults have linked longer time spent in weakly-connected state to lower score in mid- and early-life cognitive activities (Lee et al., 2019, Marques et al., 2016) and poorer cognitive reserve and higher risk of dementia (Dautricourt et al., 2022). The patients with cognitive impairment (Díez-Cirarda et al., 2018, Du et al., 2020) and depression (Yao et al., 2019) displayed increased dwell time in the weakly-connected state than the strongly-connected state. Therefore, the FNC transition we observed, from weakly- to strongly-connected states, may suggest that with development, the brain functional network becomes more and more optimized to support more efficient information exchanges and more complex cognitive and behavioral functions.

### 4.3 The effect of preterm on FNC dynamics

Recent studies from our group have shown that preterm-born infants exhibit alterations in both structural and functional architecture (Li et al., 2022, Liu et al., 2021, Zheng et al., 2023). Here, we extended the works to find that preterm birth also significantly affected dynamic FC among RSNs, specifically resulting in decreased variances of FNC. This may indicate poorer connection patterns possibly underlying less information communications among different networks (Dong et al., 2019) and imply an impairment effect of preterm birth on flexibility in the FNCs of brain networks (Wen et al., 2020). Preterm birth disturbed the brain’s functional organizations in RSNs and caused their atypically slow development (Arimitsu et al., 2022), which may be associated with the incompletion of neuronal migration due to premature removal from the maternal environment (Vasung et al., 2019). Moreover, we also observed that preterm infants preferred to spend a longer time than term infants in the weakly-connected state. This is consistent with a recent report that the preterm group dwelled significantly longer in a state characterized by generally weak connectivity between networks (Ma et al., 2020). Additionally, studies of dynamic FC in adults have shown that the dwell time in a weakly-connected state is associated with cognitive and intellectual impairment in neuropsychiatric disorders (Fiorenzato et al., 2019, Yao et al., 2019). Therefore, the prolonged time preterm infants spent in the weakly-connected state may indicate abnormal dynamic development in the functional architecture of brain networks. In the present study, the preterm and term infants with matched PMA have significant differences in both GA and PNA, and thus the dFNC differences between them may be due to exogenous influences and extrauterine experience preempting age-typical neurodevelopment (Cook et al., 2023).

### 4.4 Limitation

The present study has several limitations. First, accumulating evidence has indicated that human brain dynamics are important for predicting cognitive functions (Bassett and Mattar, 2017, França et al., 2022). We did not examine the relationship between the dFNC and later neurodevelopmental outcomes due to the lack of behavioral rating scales. Second, the sliding-window approach has been widely used in many previous studies of dynamic FC analysis, but it requires selecting arbitrary parameters like window and step sizes. Although the present study used different parameters to validate reliability, future studies could benefit from the choice of a time-resolved approach like Leading Eigenvector Analysis (LEiDA) (Lord et al., 2019) in brain dynamics analysis. Third, our analysis treated brain state occurrence in an independent fashion, i.e., without memory. Future research could develop metrics that account for how previous brain states influence subsequent states. Finally, studies in adults indicate that the dynamic FC is structurally constrained by white matter tracts (Liao et al., 2015, Zhang et al., 2016). However, understanding how anatomical substrates (e.g., cortical morphology and white-matter structural connectivity) contribute to the development of the connectome dynamics warrants further investigation.

## 5 Conclusion

This study explored the dynamic changes in FNC among RSNs during early infancy. We identified a transition pattern where FNC tended to shift from a weakly- to a strongly-connected state with increasing GA and PNA. Additionally, we observed an age-dependent development pattern in the connectome, where GA and PNA showed distinct effects on the variance of FNC over time during MRI scans. Moreover, preterm-birth decreased the flexibility of FNC across RSNs, resulting in prolonged time spent in the weakly-connected state compared to the strongly-connected state in early brain functional connection. These findings suggest an age-related temporal variance in functional development at the large-scale network level during early stage, and provide a new insight into understanding the early brain development.

## CRediT authorship contribution statement

**Dan Wu:** Writing – review & editing, Supervision, Methodology, Investigation, Conceptualization. **Mingyang Li:** Supervision, Methodology. **Yihan Wu:** Methodology, Investigation, Data curation. **Ruolin Li:** Writing – original draft, Visualization, Software, Methodology, Investigation, Formal analysis. **Zhiyong Zhao:** Writing – review & editing, Writing – original draft, Visualization, Supervision, Software, Methodology, Investigation, Formal analysis, Conceptualization.

## Declaration of Competing Interest

The authors declare that they have no known competing financial interests or personal relationships that could have appeared to influence the work reported in this paper.

## Data Availability

The data used in this study, including the imaging and collateral data, were obtained from the dHCP project. This project is publicly available and open-access. The data used in this study were included in the second dHCP data release in 2019. To access these data, interested parties can register at https://data.developingconnectome.org/.

## Footnotes

Appendix A Supplementary data associated with this article can be found in the online version at doi:10.1016/j.dcn.2024.101496.
