# Supplementary material for: State-dependent inter-network functional connectivity development in neonatal brain from the developing human connectome project
Source: Dev Cogn Neurosci. 2024 Dec 12;71:101496. doi: 10.1016/j.dcn.2024.101496 (PMC11720898; doi:10.1016/j.dcn.2024.101496)
Supplement: Supplementary file 1 — Supplementary material [file mmc1.docx]

**Supplementary Material**

**Table S1. The effect of age on FNC among RSNs at the whole-brain and network levels by regression analysis in whole term samples.**

|  | R | P (FWE correction) |
| --- | --- | --- |
| **Whole-brain level** |  |  |
| sFC - GA | -0.155 | 0.016 |
| dFC - GA | 0.109 | 0.090 |
| sFC - PNA | -0.015 | 0.816 |
| dFC - PNA | -0.005 | 0.944 |
| **Network level** |  |  |
| *sFC - GA* |  |  |
| lMN | -0.246 | 1.125e-04 |
| SSN | -0.195 | 0.002 |
| pPN | -0.255 | 6.114e-05 |
| *dFC - GA* |  |  |
| mMN | -0.225 | 4.218e-04 |
| SSN | -0.226 | 3.880e-04 |
| TN | 0.201 | 0.002 |
| *sFC - PNA* |  |  |
| rAN | -0.257 | 5.261e-05 |
| lPN | 0.263 | 3.399e-05 |
| FPN-I | -0.241 | 1.495e-01 |
| PFN | 0.275 | 1.380e-05 |
| *dFC - PNA* |  |  |
| lVN | 0.278 | 1.097e-05 |

mMN/lMN: medial/lateral motor network; SSN: somatosensory network; rVAN: right visual association network; FPN: frontoparietal network; pPN/lPN: posterior/lateral parietal network; TN: temporoparietal network; PFN: prefrontal network; rAN: right auditory network; lVN: left visual network.

**
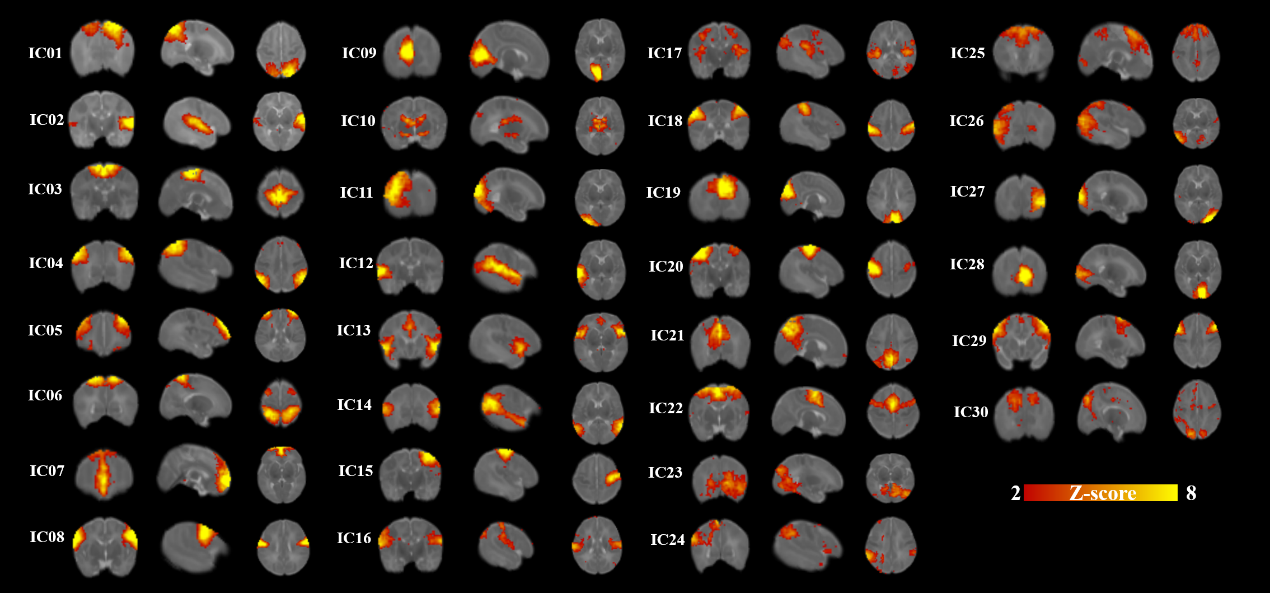
**

**Figure S1 Display of 30 components obtained from ICA.**


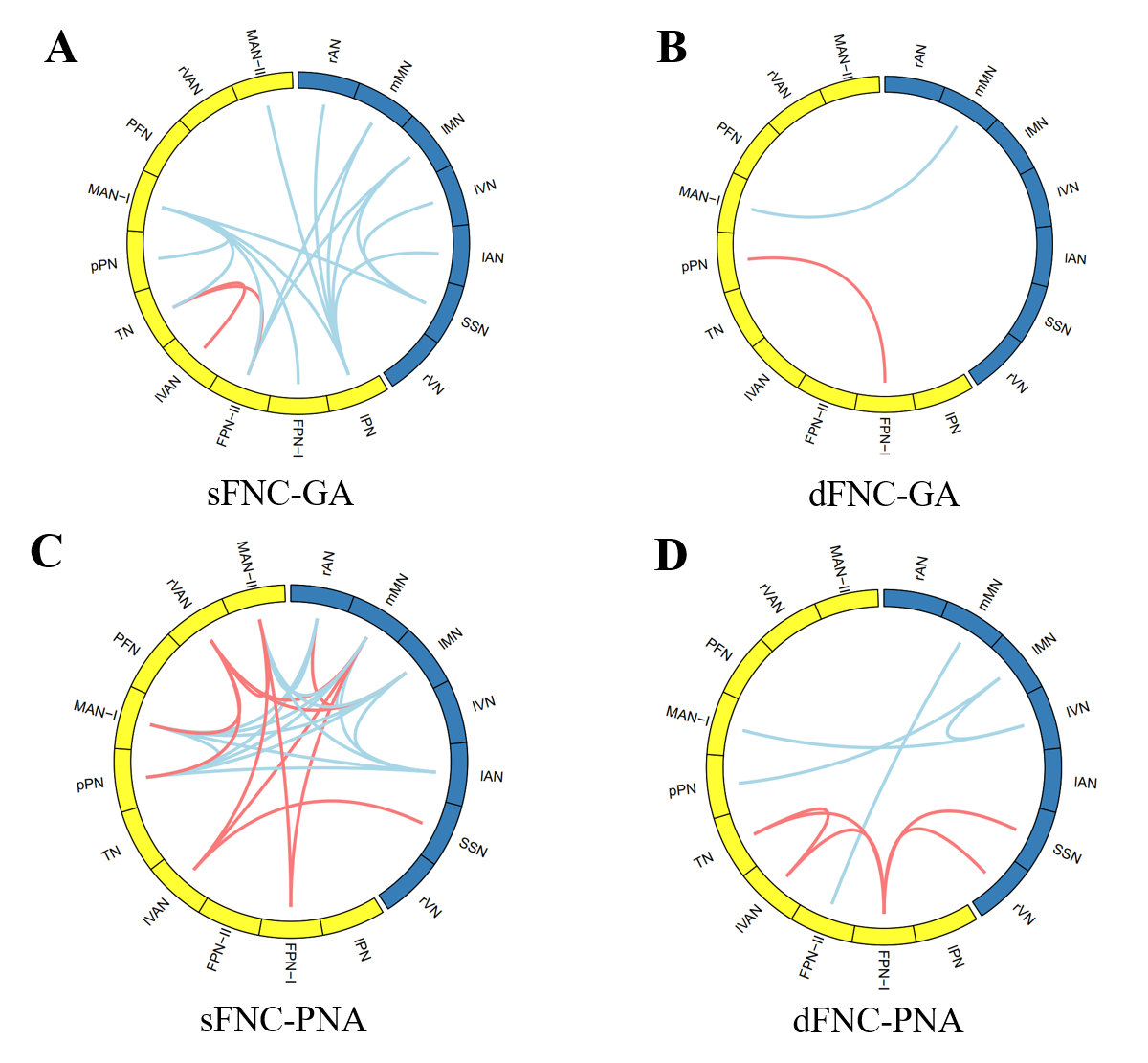


**Figure S2. The effect of age on FNC among RSNs at the connection level by regression analysis in whole samples.** In each chord diagram, the yellow and blue of the nodes represent association and primary networks, respectively. Red and blue lines represent positive and negative correlations, respectively. mMN/lMN: medial/lateral motor network; SSN: somatosensory network; rVAN/lVAN: right/left visual association network; FPN: frontoparietal network; pPN/lPN: posterior/lateral parietal network; TN: temporoparietal network; MAN: motor association network; PFN: prefrontal network; rAN/lAN: right/left auditory network; rVN/lVN: right/left visual network.
